# Sedation with Intranasal Dexmedetomidine in the Pediatric Population for Auditory Brainstem Response Testing: Review of the Existing Literature

**DOI:** 10.3390/healthcare10020287

**Published:** 2022-02-01

**Authors:** Pasquale Marra, Arianna Di Stadio, Vito Colacurcio, Alfonso Scarpa, Ignazio La Mantia, Francesco Antonio Salzano, Pietro De Luca

**Affiliations:** 1 Department of Medicine, Surgery, and Dentistry, University of Salerno, 84084 Salerno, Italy; pasquale.marra.m178@gmail.com (P.M.); alfonsoscarpa@yahoo.it (A.S.); frsalzano@unisa.it (F.A.S.); dr.dlp@hotmail.it (P.D.L.); 2 Department GF Ingrassia, University of Catania, 95123 Catania, Italy; ilamantia@unict.it; 3 Department of Otolaryngology Head and Neck Surgery, University Hospital of Modena, 41124 Modena, Italy; v.colacurcio@gmail.com

**Keywords:** intranasal dexmedetomidine, auditory brainstem response, ABR, sedation

## Abstract

Background: Intranasal dexmedetomidine (IN DEX) is a relatively new sedative agent with supporting evidence on its efficacy and safety, which can be used for procedural sedation in children, and could have a major role in auditory brainstem response testing, especially in the case of non-cooperative children. The goal of this systematic review is to assess the role of IN DEX in ABR testing, evaluating the reported protocol, potential, and limits. Methods: We performed a comprehensive search strategy on PubMed, Scopus, and Google Scholar, including studies in English on the pediatric population, without time restrictions. Results: Six articles, published between 2016 and 2021, were included in the systematic review. Sedation effectiveness was high across the studies, except for one study; 3 μg/kg was the dosing most often used. A comparison group was present in three studies, with oral chloral hydrate as the drug of comparison. Adverse effects were rarely reported. Conclusion: This systematic review showed how IN DEX can represent an adequate sedative for children undergoing ABR testing; larger and more rigorous trials are warranted in order to recommend its systematic utilization.

## 1. Introduction

Auditory brainstem response (ABR) is a crucial screening test for hearing loss in children, representing the gold standard for objective hearing evaluation in infants and toddlers [1]. ABR testing require in children the administration of a safe and stable sedative drug to avoid artifacts; in fact, babies cannot be calm enough to allow the test or unable to sleep, while children cannot be cooperative as in case of subjects with behavioural or developmental disabilities [2]. Children’s sedation is not a mundane practice [3] but it requires trained personnel and adequate facilities to be performed [4]. Traditional sedatives, like chloral hydrate, benzodiazepines, and barbiturates, could pose some risks, like vomiting, respiratory depression, and even death [3,5]. With the discontinuation in the production of chloral hydrate in several countries [6], midazolam appears to currently be the most used sedative in the pediatric population [7], but other safer options are emerging [8]. Among them, dexmedetomidine is a relatively new anxiolytic and sedative drug with a selective agonist activity for alpha-2 adrenergic receptors in the central nervous system [9]. It has been widely used in pediatric care, often as an adjunctive drug, to induce sedation, manage pain, and facilitate many procedures, both diagnostic and therapeutic [10]. The advantage of Dexmedetomidine (DEX) appears to be its stronger safety profile, including the lack of negative respiratory effects [4]. It can be administered intravenously, but the intranasal (IN) route is also an option, with the advantage of avoiding the stress and pain associated with IV catheter insertion in a non-cooperative patient [11,12].

Over the last decade, several clinical trials have been conducted on IN DEX, inquiring about its role in many pediatric procedures, ranging from CT [13] to echocardiography [14] to ophthalmic examination [15]. Systematic reviews have also been written on the topic of procedural sedation with IN DEX [7,16,17]. However, no literature review has been produced about its specific utilization in ABR testing; while being a reasonable option, this use case remains sparse and probably overlooked, with the absence of a clear standard protocol [12].

Advancing the knowledge about IN DEX in ABR testing could represent an invaluable opportunity for audiologists and anesthesiologists who seek to accomplish an adequate ABR execution in non-collaborative pediatric patients. The aim of this review is to shed some light on the utilization of IN DEX in ABR testing, evaluating the reported protocol, potential, and limits.

## 2. Materials and Methods

**Figure 1:**
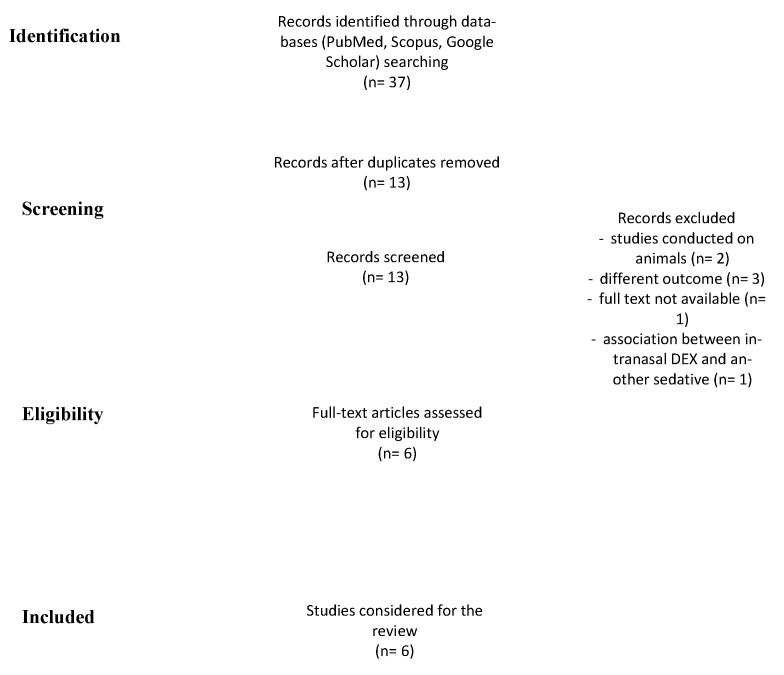
PRISMA chart shows the method used for doing this systematic review.

This study was performed in accordance with the Preferred Reporting Items for Systematic Reviews and Meta-analysis (PRISMA) guidelines (Figure 1) and was registered to PROSPERO with n 303507 (waiting for definitive approval). Because of the nature of this work, the Institutional Review Board approval was not requested.

### 2.1. Search Strategy

A comprehensive search strategy, developed in partnership with a medical librarian, was performed on PubMed, Scopus, and Google Scholar without time restrictions (1 January 1900–19 December 2021). The keywords used were: “intranasal”, AND “dexmedetomidine”, AND “auditory brainstem response”. Only articles in the English language were considered for the analysis.

Two independent investigators reviewed the articles extracted from the literature review. Duplicates were removed, then each reviewer singularly filled in an Excel data sheet (Microsoft Corporation, Redmont, WA, USA) including information extracted from the articles in agreement with inclusion and exclusion criteria (see below). The datasheets were compared, and disagreements were debated until complete agreement of both researchers. Only papers that received full consensus were considered. PRISMA guidelines were followed to conduct the systematic review and the full list of references was screened for potentially relevant articles. Selected articles were read in full to assess the study objectives and the level of evidence.

### 2.2. Inclusion and Exclusion Criteria

Inclusion Criteria: patients (0–18 years) underwent auditory brainstem response with IN DEX, written in the English language, with full-text available.

Exclusion Criteria: articles with a lack of information about the use of dexmedetomidine, articles that report the use of IN DEX in association with other sedatives, and case reports.

### 2.3. Risk of Bias Assessment

The National Institutes of Health’s (NIH) quality assessment tools were used to assess the risk-of-bias checklists due to the different study designs [18]. The rating of each study was categorized as: poor, fair, or good (i.e., unbiased and fully described). The two authors independently gave a score to each article and any disagreement was resolved by direct comparison among the researchers (Table 1).

## 3. Results

### 3.1. Study Selection

A total of 37 records were identified (Figure 1). After the removal of duplicates and abstract evaluation, 24 articles were excluded. Thirteen articles matched the inclusion/exclusion criteria. Seven articles were excluded (*n* = 2, studies conducted on animals; *n* = 3 high risk of bias due to different outcomes; *n* = 1, no full-text available; and *n* = 1, IN DEX in association with another type of sedative) and the remaining six were included in the systematic review. The articles identified the use of IN DEX for pediatric ABR tests. All studies were published over a period of five years, between 2016 and 2021.

### 3.2. Study Characteristics

Six full-text articles were identified [4,6,12,19,20,21] (Table 2); we identified three retrospective works, two double-blind randomized controlled trials, and one prospective no-randomized work. Three studies were conducted in the United States of America, one in China, one in Singapore, and one in the United Kingdom. 454 patients were evaluated in the IN DEX group (358 male, 78.8%; and 89 females, 21.2%; age range 0.2–114 months); the hearing function was always (100%) tested by ABR. A comparison group was present in three studies, with oral chloral hydrate as the drug of comparison.

### 3.3. Effectiveness of Sedation

Different definitions of sedation effectiveness were used across the studies, mainly based on subjective or qualitative evaluations. Reynolds [20] showed a high number of satisfactory sedation from a single dose (89%); the same author in a prospective and randomized study [6], showed the ability to complete ABR examination with one sedative dose was 91%. Similarly, Baier et al. [19] found a 90% success rate by the first dose, Godbehere et al. [4] identified a 100% success rate, and Fan et al. [12] reported 83% sedation success. Conversely, Li et al. [21] described a lower success rate (64.3%) compared with the results of other authors. Finally, IN DEX was deemed comparable to chloral hydrate in three studies [6,20,21].

### 3.4. Protocol of Use

The optimal dose ranged from 2 to 4 μg/kg; 3 μg/kg was the dosing most often used. Three studies [6,19,20] reported a maximal dose of 100 μg. Atomizer devices for intranasal nebulization were used in all the studies.

## 4. Discussion

IN DEX has an established role in procedural sedation for the pediatric population, including ABR testing, although the number of studies focusing on its use exclusively for ABR is still limited. Because of the lack of homogeneity among studies and the use of different subjective methodologies, it was not possible to evaluate the efficacy of sedation in a quantitative way. However, most of the studies included in our review showed a high percentage of success. The results extracted from the papers we reviewed overlap other similar studies, in which adequate sedation has been achieved in a high percentage of the patients undergoing non-painful procedures [7], such as ABR. Only Li et al. showed a controversial result [21] with 64.3% success rate. This difference might be related to the characteristics of the population included in the study (patients with autism spectrum disorder only), the assessment used for evaluating sedation, the nature of the study, the small number of subjects undergoing ABR only, and the choice of using a full profile of latency-intensity function and highest intensity stimulus (90–100 decibels) in all children. The author speculated that the level of sedation with DEX could be too shallow for such stimulation and found that adding oral midazolam (0.2 mg/kg) could improve the results (87.5% of success rate) but prolong the awakening time with delayed hospital discharge.

Reynold [6,20] compared DEX to oral chloral hydrate in two different studies and found similar efficacy. DEX was safer than chloral hydrate [7]; the latter could present a carcinogenetic risk [22]. Moreover, chloral hydrate is no longer approved by the Food and Drugs Administration. In literature, there are no available studies comparing DEX and other alternatives like midazolam, propofol, ketamine, and emerging options, like melatonin [8], to induce sleep in patients who undergo ABR. In addition, the studies performed using these treatments are not strongly designed, i.e., large double-blind randomized trials are warranted.

The studies were quite homogenous in terms of the administered dose, most of the authors used 3 μg/kg to induce sleep. Fan et al. [12], using this dosage, reported adequate sedation for ABR but noted significant differences in weight and age between babies who were adequately sedated by IN DEX only and the ones who did not; however, adequate sedation was obtained more frequently in ABR than other procedures investigated in this study, suggesting the IN DEX to be particularly suitable for this use case. A single dose appears to be sufficient to achieve sedation in the majority of cases, and multiple administrations can be given, if necessary, without adverse effects; the maximum dosage more often reported is 100 μg. The drug can be administered indifferently in one or both nostrils. Several studies [7,9,10] have highlighted that IN DEX is better than intravenous or oral administration since it does not require IV access or cooperation of the patient to swallow and it is easier to administer. The benefits to the patients are less pain, because no intravenous access is needed; IN administration is better tolerated and lack of discomfort in nose, nasopharynx [21]; this is because DEX is tasteless and odourless, whereas midazolam is reported to be unpleasant and irritating to the nasal mucosa [6]. The IN administration by mucosal atomization device was used in all the studies included in our review and had good patients’ compliance.

Bioavailability following IN administration appears to be more consistent than the oral route since it avoids the first-pass metabolism, and the delivery is more direct and therefore more reliable [6]. IN DEX reaches peak plasma concentrations in 46 min at 1 μg/kg dosing and 47 min at 2 μg/kg and has absolute bioavailability of 83.8% as reported by Miller et [22]; the authors found that the mean arterial plasma concentrations of dexmedetomidine for the lower dose was reached within 20 min, while doubling the dose allowed to reach plasma concentration within 10 min [22]. These characteristics allow to rapidly perform ABR. Moreover using nasal administration the drug has a half-life lower than 40 min that allows a faster return to normal consciousness, extremely important for children; and, as shown by Reynolds et al. [6], this rapid pharmacokinetic of DEX could reduce parental supervision after the test, and risks of falls because child remains partially sedated. DEX has a gradual action and seems to induce sedation similar to natural sleep, i.e., lighter at the beginning [12]; this particular induction, makes more convenient the use of DEX in the facilities where the ABR will be performed. DEX, exactly as spontaneous sleep activates the endogenous sleep pathway [23], that is desirable in a test like ABR; the action at this level mimics the natural condition without a negative impact on the ABR recording exactly as possible by using a combination of natural elements [8].

The safety profile was not primarily investigated in our work; however, in the articles included none of the authors reported major adverse events, suggesting that this is a safe drug. In only one case, an episode of respiratory distress in a child suffering from laryngomalacia and affected by a genetic disorder [20] The latter could be caused by the floppy airway in laryngomalacia which collapse more than normal (reduction of the tone in the upper airways) following sedation. Hence, a detailed history on episode of sleep apnoea or any airway issues should be collected when DEX is considered as sedative drug.

DEX safety is discussed and analyzed in other studies, where it is reported to have less impact on airway and respiratory function [7] and might have neuroprotective capacity, as shown in animal studies [17]. DEX can negatively affect heart rate and blood pressure [24], so careful monitoring of blood pressure and oxygen saturation must be done. Its use should be avoided in patients with hypotension, bradycardia, or other cardiac problems, in which the use of nutraceuticals would be better [8]. Godbehere et al. [4] proposed that ABR with IN DEX could be performed in a side room with adequate monitoring facilities and trained staff, and not in a surgical theatre, due to the safety of the drug. This has the added benefits of sparing the theatre for more urgent procedures, putting less time pressure on the audiologist, and reducing anxiety for children and parents. Moreover, performing the ABR outside the theatre allows to reduce the electrical interference which could negatively affect the ABR results [4].

Regarding the specific effect that DEX could have on ABR, no data were found. DEX is reported to not influence the brainwaves activity during EEG [19]; however, little is known on its effects, if any, on ABR waves. Qualitative differences in ABR results obtained with different sedatives should be further investigated and the influence of dexmedetomidine should be excluded. The effect of different timing and a wider range of stimulus intensity and frequency should be also evaluated. These investigations will necessarily require the active involvement of trained audiologists.

To note that none of the studies included in this review used objective and validated methods, such as Bispectral Index (BIS) monitoring, to quantify the level of sedation.

### Limits of IN DEX

Despite the promising promises, the use of IN DEX seems to have some limitations. It should be noted that IN DEX still requires some degree of cooperation from the patient: the child may be bothered by both the context and the intranasal device, and resist instillation. Also, in the case of a cold or other pathology of the nasal mucosa, the absorption may not be as effective. In addition, the intranasl administration can favor ripetute sneezing and cannot ensure the full potential of the instilled drug. Moreover, even when a specific device (atomiser) is used, part of the inhaled nebulized DEX may be exhaled.

## 5. Conclusions

Although DEX seems quite ideal for performing ABR test (rapid onset, favorable side effect profile, effective in inducing sedation, and rapid patient recovery), the drug is not the standard of choice, as still much is unknown about this drug and its utilization during this type of procedure. This review highlighted the need of high-quality studies about the IN DEX in ABR. Randomized-controlled trials are needed to better characterize the onset of effect, recovery time, and adequacy of sedation during ABR using validated scales.

## Figures and Tables

**Table 1:** Summary of studies included in the systematic review.

References	Design of the Study	Overall Quality Ranking Consensus
Reynolds et al. (2015)	Prospective randomized double blind trial	Good
Bayer et al. (2016)	Retrospective	Fair
Reynolds et al. (2016)	Retrospective	Fair
Li et al. (2019)	Prospective randomized double blind trial	Good
Godbehere et al. (2021)	Prospective	Fair
Fan et al. (2021)	Retrospective	Fair

**Table 2:** Characteristics of studies exploring the effect of intranasal dexmedetomidine protocol for patients for pediatric ABR tests.

References, Nation, Year	Type of Study	Sample Size	Age (Median, Range; Months), Weight (Median, Range; Kilos), Gender	Dexmedetomidine Protocol, Device Used	Effectiveness of Sedation Definition	Control Group	Conclusion
						(Age, Median, Range, Months; Gender; Type of Intervention)	
Reynolds et al., United States of America, 2015	Prospective, randomized, double-blind	44	23.3 (19.5–27.2), months;	3 μg/kg	State that allowed the audiologist to place ABR electrodes.	25.6 (22.0–29.0), months	DEX is as effective as CH, higher incidence of testing completion with a single dose, shorter time to desired sedation level, more patients reported to return to baseline activity on the same day
			12.3 kg (11.2–13.4 kg)	Max dose = 100 μg		27 M/14 F	
			23 M/21 F	MAD Nasal™ needle-free intranasal drug delivery system (Teleflex Medical, Research Triangle Park, NC)		50 mg/kg chloral hydrate (CH), with saline placebo for intranasal administration	
Baier et al., United States of America, 2015	Retrospective	52	3.6 (2.4–8.4), months	2.5–3 μg/kg	First time the patient’s	NA	IN DEX is an effective and non-invasive method of sedating children for ABR
			6.7 kg (5.6–8.8 kg)	Max dose = 100–150 μg	level of consciousness was noted to be ‘sedated’ by the attending nurse		
Reynolds et al., United States of America, 2016	Retrospective	100	27.1 ± 15.6 months	4 μg/kg	ability to complete the examination with a single dose of medication	28.6 ± 18.6 months	IN DEX provides effective sedation for ABR examinations, with the benefits of an ability to begin the test sooner and complete the examination with a single dose, in addition to a decreased incidence of hypoxemia
			12.9 ± 4.0 kg	Max dose = 100 μg		110 M/90 F	
			67 M/33 F			Oral CH (dose not specified)	
				MAD Nasal needle-free intranasal drug-			
				delivery system			
		
Li et al., China, 2019	Prospective, randomized, double-blind	14	35.0 (28.0–44.8), months	IN DEX 3 μg/kg and buccal placebo	UMSS (University of Michigan Sedation Score), of 2–4 and completion	34 (28.0–46.0), months	Combination of IN DEX and buccal midazolam was associated with higher sedation success when compared to IN DEX
			122 M/14 F	Max dose = NA	of ABR examination	130 M/9 F	
				MAD NasalTM, Telefex Incorporated, USA		IN DEX at 3 μg/kg plus buccal midazolam at 0.1 mg/kg mixed with simple syrup	
			note: autism spectrum disorder diagnosis				
Godbehere et al., United Kingdom, 2020	Prospective	29	38.55 (12–114), months	IN DEX at 3 μg/kg	Child no longer sensitive to touch	NA	IN DEX could be used successfully to administer safe sedation to all 29 children undergoing an ABR in a ward environment as opposed to theatre
			20 M/9 F	Max dose = 100 μg			
				Atomizer device			
Fan et al., Singapore, 2021	Retrospective	12	20.0 (10.5–26.0)	IN DEX was administered at a dose of 2 to 4 μg/kg	Completion of procedure	NA	IN DEX is effective for procedural sedation for pediatric patients. The most important predictor for sedation success was indication of sedation and duration of procedures
			7 M/5 F	Max dose = NA	or investigation with dexmedetomidine as the only agent.		
				Mucosal atomizer device			

## Data Availability

Not applicable.
